# Fast electron transport dynamics and energy deposition in magnetized, imploded cylindrical plasma

**DOI:** 10.1098/rsta.2020.0052

**Published:** 2020-12-07

**Authors:** D. Kawahito, M. Bailly-Grandvaux, M. Dozières, C. McGuffey, P. Forestier-Colleoni, J. Peebles, J. J. Honrubia, B. Khiar, S. Hansen, P. Tzeferacos, M. S. Wei, C. M. Krauland, P. Gourdain, J. R. Davies, K. Matsuo, S. Fujioka, E. M. Campbell, J. J. Santos, D. Batani, K. Bhutwala, S. Zhang, F. N. Beg

**Affiliations:** 1 Center for Energy Research, University of California San Diego, La Jolla, CA 92093-0417, USA; 2 Laboratory for Laser Energetics, University of Rochester, Rochester, NY 14623, USA; 3 E.T.S.I. Industriales, Universidad Politecnica de Madrid, Madrid 28040, Spain; 4 Office National d’Etudes et de Recherches Aérospatiales (ONERA), Palaiseau 91123, France; 5 Sandia National Laboratories, Albuquerque, NM 87185, USA; 6 Department of Physics and Astronomy, University of Rochester, Rochester, NY 14627, USA; 7 General Atomics, San Diego, CA 92186, USA; 8 Extreme State Physics Laboratory, University of Rochester, Rochester, NY 14627, USA; 9 Institute of Laser Engineering, Osaka University, Suita, Osaka 565-0871, Japan; 10 Université de Bordeaux-CNRS-CEA, CELIA UMR, 5107 33400 Talence, France

**Keywords:** ICF, fast electrons, compression

## Abstract

Inertial confinement fusion approaches involve the creation of high-energy-density states through compression. High gain scenarios may be enabled by the beneficial heating from fast electrons produced with an intense laser and by energy containment with a high-strength magnetic field. Here, we report experimental measurements from a configuration integrating a magnetized, imploded cylindrical plasma and intense laser-driven electrons as well as multi-stage simulations that show fast electrons transport pathways at different times during the implosion and quantify their energy deposition contribution. The experiment consisted of a CH foam cylinder, inside an external coaxial magnetic field of 5 T, that was imploded using 36 OMEGA laser beams. Two-dimensional (2D) hydrodynamic modelling predicts the CH density reaches $9.0\;{\text{g cm}}^{-3}$, the temperature reaches 920 eV and the external B-field is amplified at maximum compression to 580 T. At pre-determined times during the compression, the intense OMEGA EP laser irradiated one end of the cylinder to accelerate relativistic electrons into the dense imploded plasma providing additional heating. The relativistic electron beam generation was simulated using a 2D particle-in-cell (PIC) code. Finally, three-dimensional hybrid-PIC simulations calculated the electron propagation and energy deposition inside the target and revealed the roles the compressed and self-generated B-fields play in transport. During a time window before the maximum compression time, the self-generated B-field on the compression front confines the injected electrons inside the target, increasing the temperature through Joule heating. For a stronger B-field seed of 20 T, the electrons are predicted to be guided into the compressed target and provide additional collisional heating.

This article is part of a discussion meeting issue ‘Prospects for high gain inertial fusion energy (part 2)’.

## 1. Introduction

The fast ignition (FI) concept [1] for inertial confinement fusion (ICF) proposes to separate the compression and ignition phases of a fuel pellet. This scheme proceeds by first compressing the pellet with an array of laser beams, and then rapidly heating a localized spot on the compressed plasma with a short peta-watt laser pulse, to ignite a fusion reaction that will spread to the rest of the plasma. It reduces the complexity and expense of the laser optics needed for the compression stage, and is predicted to reduce the total energy needed to achieve ignition. Consequently, a higher gain is expected with the FI scheme, which would burn more fuel mass with less compression energy. In the electron FI scheme, the weaker compression is compensated by the use of a high intensity (greater than 10^18^ W cm^−2^) laser which produces a relativistic electron beam [2,3] that heats the compressed fuel plasma to initiate ignition. It has been estimated that the electron beam with kinetic energies of $\langle \varepsilon_{h}\rangle =1-2\;\text{MeV}$ needs to deposit ≈20 kJ into a 20 μm radius lateral hot spot in the compressed core to trigger ignition [4]. However, the generation and transport of the fast electron beam from its generation point to the dense core is complex and raised several issues [5,6], mainly due to the beam divergence [7]. A major challenge is therefore to ensure a guided electron beam propagation within the FI integrated target before it reaches the fuel core.

Downscaled FI experiments have been performed in spherical geometry, first at the Institute of Laser Engineering (ILE) at Osaka University in Japan [8–10] and then at the Laboratory for Laser Energetics (LLE) at the University of Rochester [11]. More recently, some experiments have developed a platform on the OMEGA laser facility [12,13] to diagnose the fast electron transport and its energy deposition using a Cu dopant and X-ray diagnostics. In these experiments, a gold cone was used to inject the fast electrons into a spherical imploding target. Experimental data showed that a significant number of electrons deposited their energy around the cone wall rather than in front of the tip, suggesting that new schemes are warranted to mitigate relativistic electron divergence [12].

An external B-field can reduce the divergence of the electron beam, as demonstrated in planar geometry [14], and could improve the energy deposition efficiency of the fast electrons. To guide or confine a MeV-energy electron beam, one needs to magnetize the target at several hundreds of Tesla so that the electron Larmor radius becomes smaller than the electron beam size. A set of numerical simulations of FI cone target implosions, with artificially imposed axial B-field were performed by Strozzi *et al.* [15]. It has been shown that a 5 kT axial B-field resulted in reducing the required fast electron energy from approximately 1 MJ to approximately 130 kJ. Recently at ILE, Osaka University, the magnetized fast isochoric heating experiment demonstrated a factor of 2 enhancement of laser-to-core energy coupling using an external kilo-Tesla-level B-field generated by a laser-driven capacitor coil in a spherical geometry [16,17]. The B-field configuration through the implosion and also the electron transport in the resultant compressed target have been analysed numerically in [18–20]. In the context of a FI integrated target, the magnetic mirror effect at the cone injection caused by the B-field compression at the cone tip would also reduce the coupling of the relativistic electrons to the fuel.

Considering these challenges, a cylindrical geometry arises as a promising option, as it allows for compression in the radial dimension and leaves a free axis to apply the external B-field and inject the fast electrons. In this scheme, the implosion front of the cylinder is ideally parallel to the applied B-field direction. Therefore, the mirror angle of the compressed B-field becomes smaller than that in a spherical target compressed from all directions. Magnetized liner inertial fusion (MagLIF) scheme [21,22] is a recent example of a scheme based on radial compression and longitudinal heating.

Long before petawatt lasers were constructed, Sweeney *et al.* considered a class of high-gain ICF targets driven by electrons or light ions with strong external B-fields in cylindrical compression [23]. According to their suggestions, energy gains of 20–40 in the compressed density of 10 g cm^−3^ are expected using 1 MeV electron beam injection with an intensity of 45 TW cm^−2^ and initial B-fields of 30–60 T.

Fast electron transport experiments in cylindrical targets without an external B-field have been conducted [24–27], highlighting the importance of a self-generated B-field associated with the resistivity gradient on the implosion front, which can guide the electron beam under the right conditions. This scheme is also compatible with an axial B-field to guide the high-energy electron beam in FI, in which both self-generated B-field and compressed external B-field can reduce the electron divergence. Improving the electron beam confinement along the propagation axis would significantly raise the electron energy flux deep into a target, and many subsequent physical mechanisms would benefit from those developments, such as secondary sources of particles [28], hard X-rays [29] and gamma-ray [30] emission yields, or isochoric heating of dense matter [31].

Several challenges remain for the realization of a FI-like concept in cylindrical geometry related to the accurate description of the magneto-hydrodynamics of the implosion, such as the magnetization of the dense core, and the optimization of fast electron energy deposition into the core.

In our experiment, the compression of a cylindrical target with an external seed B-field of 5 T was achieved using 36 beams of the OMEGA-60 laser. Then, the high-intensity OMEGA-EP laser irradiated a foil end-cap of the cylinder to produce relativistic electrons to heat the imploding cylindrical plasma. A multi-stage simulation scheme was undertaken to describe the multi-scale physics from implosion to electron transport inside the cylinder. The implosion of the cylinder was modelled using the two-dimensional (2D) FLASH code, and the relativistic electron source was simulated using a 2D fully relativistic particle-in-cell (PIC) code. Then, three-dimensional (3D) PIC-hybrid simulation using both previous results calculated the electron transport inside the cylinder target with the compressed external B-field. From the multi-stage simulation analysis, motivated by the complex experimental results, we reveal the conditions for which a self-generated B-field and an external B-field can guide the fast electrons before and after the compression time.

This paper is organized as follows. Section 2 shows the experimental set up, providing details about the cylindrical target, the laser drivers and the external B-field. The experimental data are shown in §3. The simulation results for the hydrodynamic implosion and the fast electron generation are presented in §4a,b, respectively. The hybrid-PIC simulations of electron transport in the imploded plasma are presented in §5. First, the transmitted electron spectra are shown in the context of the experimental results. Then the mechanisms of the high-energy electron transport and resulting energy deposition before and after the maximum compression are discussed. Finally, the dependence on external B-field strength is presented in §6. The conclusions of this paper are summarized in §7.

## 2. Experimental set up

**Figure 1.**
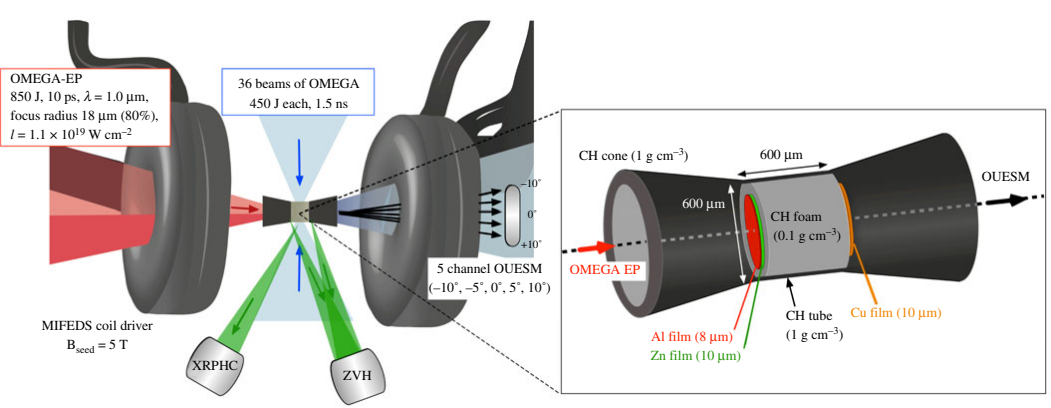
Schematics of the experimental set up, including main diagnostics (XRPHC, ZVH and OUESM) and B-field pulsed source (5 T), as well as the laser drivers’ parameters of OMEGA-60 and OMEGA-EP. The enlarged figure illustrates the dimensions of the CH cylinder foam (0.1 g cm^−3^) equipped with CH cone target (density: 1.0 g cm^−3^, thickness: 205 μm, length: 950 μm and angle: 11.9°) and high-Z foils at the front (Al, Zn) and at the rear side (Cu). The coil diameter was 4.4 mm, and each MIFEDS coil was located at ±4.2 mm from the midplane of the cylinder target. The distance between the centre of the cylinder target and the OUESM was set to 380 mm. (Online version in colour.)

The experiment was carried out at the OMEGA laser facility using 36 of the 60 OMEGA long pulse lasers to compress a cylindrical target while the OMEGA-EP short-pulse laser was used to generate a beam of fast electrons along the cylinder axis to rapidly heat the precompressed material. A schematic of the experiment is shown in figure 1. The cylindrical target was a plastic tube filled with CH foam (0.1 g cm^−3^). The tube had a wall thickness of 20 μm, an outer radius of 300 μm and a length of 600 μm. To avoid plasma expansion from the front and rear surfaces, a solid 10 μm thick zinc foil and a solid 10 μm thick copper foil were attached to either end of the cylinder. More importantly, those two foils served as indicators of fast electrons going through the cylinder whereby K_*α*_ emission lines of Cu and Zn induced by collisions from the fast electrons were detected. In order to generate the fast electrons, the OMEGA EP beam irradiated an 8 μm thick Al layer coated on the Zn foil at the end of the cylinder facing port H7. The Al layer prevented direct interaction of the OMEGA EP beam with the Zn foil, allowing it to be an indicator of only those fast electron propagating into the foam. In addition, solid CH cones (density: 1.0 g cm^−3^, thickness: 205 μm, length: 950 μm and angle: 11.9° for the cylinder’s axis) were attached to each side of the target. The goals of the cone shields are to avoid direct irradiation on the Cu and Zn foils, as well as to avoid inflow of ablating plasma into the region in front of the foils and heating of the foils by radiation emitted from the ablation region. Each long-pulse beam irradiated the cylinder surface with an average energy of 441 J and a square pulse duration of 1.5 ns with a rise time of 50 ps (from 5% to 95% of total maximum power). The short pulse OMEGA-EP laser with the wavelength *λ* = 1.054 μm irradiated the Al foil. The intensity averaged within the *r*_80%_ spot and *τ*_(FWHM)_ duration is the laser energy *E*_*L*_ × 80% spatially enclosed ×94% Gaussian temporal shape correction factor $1/(\tau_{\left(\mathrm{FWHM}\right)}\times \pi r_{80\text{\%}}^{2})$. Using measured values from one example shot of 864 J, *τ*_(FWHM)_ = 10.6 ps, and *r*_80%_ = 18.1 μm gives an average intensity of 6.0 × 10^18^ W cm^−2^. Fitting this to a Gaussian intensity profile, the peak intensity is *I*_0_ = 1.1 × 10^19^ W cm^−2^. This was the most intense shot. The lowest intensity shot had a peak intensity of 8.4 × 10^18^ W cm^−2^. The magneto-inertial fusion electrical discharge system (MIFEDS) coils of 4.4 mm diameter were positioned on both sides (at ±4.2 mm from the midplane of the cylinder target) and generated a seed B-field of 5 T along the axis of the cylinder. The magnetic field is uniform over the dimensions of the cylinder target. The compression of the target and the beam pointing has been demonstrated in [32].

Four X-ray pinhole cameras (XRPHC) with a spectral range from 2 keV to 5 keV observed the target emission at different angles. These diagnostics provided time-integrated images with a magnification of 3.95. The time-integrated Zinc Van Hamos Spectrometer (ZVH) was configured to measure a spectral range of 7 to 10 keV, including the K-shell emission spectra from the Zn foil and the Cu foil. By measuring these lines, the amount of fast electrons flowing through the foils can be inferred. Therefore, one can relate the difference between the emissions from the front and the rear foils to the energy deposition of the high-energy electrons inside the cylinder target. In addition, relativistic electrons escaping the cylinder at its rear end were detected by the Osaka University Electron Spectrometer (OUESM) [33]. The OUESM was set to 380 mm away from the centre of the cylinder target. This diagnostic consists of five channels measuring the electron distribution with different view angles covering ±10° in 5° increments where the 0° channel is in line with the cylinder axis and OMEGA EP intended propagation axis. It has a detection range over a few tens of MeV with high-energy resolution. For each channel, a magnet with field strength 4500 G was chosen.

## 3. Experimental results

**Figure 2.**
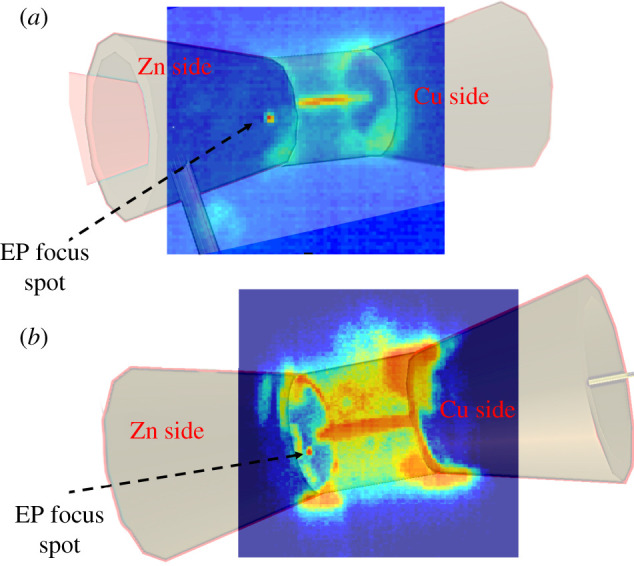
Images from two X-ray pinhole cameras showing two views of the target overlapped with 3D images of the target design. The emission point corresponding to the OMEGA EP laser irradiation is noticeably offset 100 μm away from the centre axis of the cylinder at an angle 30° from the OUESM measurement plane. (*a*) is from port H8C, 117° from the OMEGA EP propagation direction, while (*b*) is from port H13C, at 72°. (Online version in colour.)

Figure 2*a*,*b* shows time-integrated X-ray pinhole camera images obtained on the experiment from two different viewing angles, showing that the OMEGA EP focal spot position was offset laterally from the cylinder axis. The intense X-ray emission along the axis of the cylinder target, observed on both images, corresponds to the compressed plasma. It indicates heating due to the implosion. As emission brightness in the X-ray filtration band is linear with the density of radiators and strongly dependent on temperature, the image brightness strongly emphasizes emission from the dense and hot stagnation period. The measured width in the radial direction corresponds to 70 μm, while the initial cylinder diameter was 600 μm. This indicates that the cylinder target was compressed into a channel with a volume of roughly 1/75 the initial volume. On both images, an X-ray point source is observed at the cylinder front, on the Al-coated Zn foil side corresponding to the actual focusing point of the OMEGA EP laser. This source position is offset from the expected focusing position 100 μm radially from the axis of the cylinder and −30° azimuthally from the OUESM alignment plane (the sign convention for this angle follows the same sign convention as the OUESM channels’ angles seen in figure 1). This offset was consistent on all shots.

**Figure 3.**
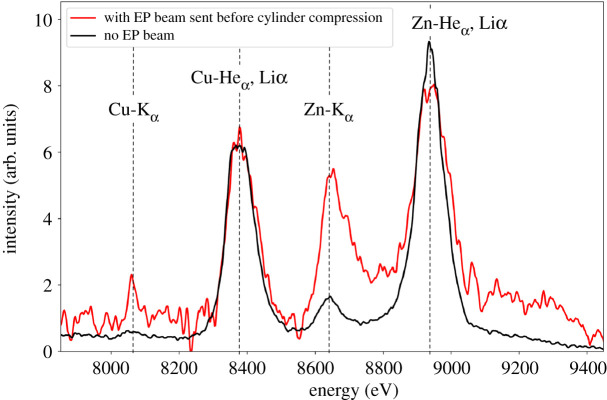
Time-integrated X-ray spectra measured by the ZVH spectrometer, without the OMEGA-EP beam (black line) and with the OMEGA-EP beam irradiating the cylinder 0.055 ns after the beginning of the 36 OMEGA beams (red line). At this time, the cylinder did not start to implode yet, it is still cold and uncompressed. (Online version in colour.)

Figure 3 presents ZVH X-ray spectra, which are integrated over the entire implosion time, for two shots: one without OMEGA-EP irradiation and one with OMEGA-EP irradiation at *τ* = 0.055 ns. *τ* is the delay between the OMEGA-60 implosion beams and the OMEGA-EP heater beam measured from the 5% nominal laser power of the respective beams when reaching the target chamber centre. In both cases, we can observe lines around 8.38 keV and 8.95 keV that correspond, respectively, to the Cu and the Zn hot lines (mainly He_*α*_, Li_*α*_), which can be attributed to heating during the implosion. The similar intensity level of the Cu and Zn hot lines indicates that the implosion for those two shots happened in similar conditions. Hence, those two shots are considered similar enough to compare the K_*α*_ emissions with or without the presence of the OMEGA-EP beam. We can see in figure 3, a significant increase of the two K_*α*_ lines when the OMEGA-EP beam is added (red curve). Cold K_*α*_ emission is a signature of the high-energy electrons driven by the OMEGA-EP irradiation, and observation of both lines confirms that some fast electrons passed through the Zn foil and CH to reach the rear Cu foil, despite the pointing offset. The Zn-K_*α*_ intensity is significantly higher than the Cu-K_*α*_ intensity, which could indicate losses in the number of high-energy electrons between the Zn and Cu foils. The possible loss mechanisms include energy deposition of the electrons inside the cylinder and lateral escape of electrons during the transport due to the pointing offset and scattering.

**Figure 4.**
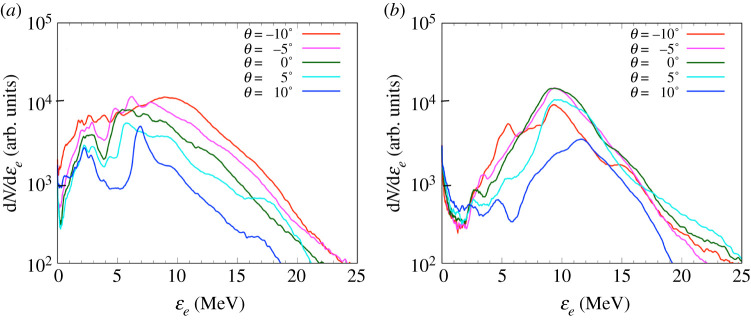
OUESM data of transmitted electron spectra for time delays *τ* = 0.00 ns (*a*) and *τ* = 1.21 ns (*b*). *N* is the measured electron number, and *ϵ*_*e*_ is the electron kinetic energy. The quantity of electrons at −10° is the highest value among all angles for the both time delays. (Online version in colour.)

Figure 4 shows the experimental transmitted electron spectra obtained from the OUESM. Spectra are shown from two shots with *τ* = 0.00 ns (a) and *τ* = 1.21 ns (b) for the five angular channels. The timing of *τ* = 1.21 ns corresponds to the time for which the CH foam reaches its maximum temperature in the experiment for $B_{\text{seed}}=5\;\text{T}$, inferred from the time-resolved X-ray diagnostic SXS (as in [32]). The comparison of this timing in the experiment with the hydrodynamic simulations and results of [32] will be discussed in §4a. At the start of the compression, *τ* = 0.00 ns, the quantity of electrons at −10° is the highest value among all channels. Signal decreases for the channels with greater angles. This asymmetry of the spectra is due to the offset of the laser irradiation.

At *τ* = 1.21 ns, the angular dependence of the spectra becomes small compared with the result at *τ* = 0.00 ns. In addition, the numbers of the transmitted electrons gradually decrease from 10 MeV to 2 MeV for all angles, and are over 10 times smaller at 2 MeV compared to 10 MeV. The lower the energy of an electron, the greater probability that it will be absorbed or deflected away from the detector by the magnetic fields. Hence, we suspect that the electrons with an energy less than 10 MeV are absorbed inside the imploded cylinder or reflected backward due to the imploded and self-generated B-fields. In conclusion, the electron spectrum data show (i) asymmetry of the electron beam angular distribution, consistent with the offset position of EP laser and (ii) a stronger reduction of the number of electrons for greater than 10 MeV, which seems consistent with an increased absorption of electrons and a magnetic mirror effect.

## 4. Simulations of the implosion conditions and fast electron source

The numerical description of the integrated fusion schemes can be broken into multi-stage simulations differentiated by the different time steps and spatial scales. First, the implosion of the cylinder was modelled using 2D axisymmetric, cylindrical simulation in (*r*, *z*) with the FLASH code, where one coordinate (*r*) corresponds to the cylinder’s radius direction and the other (*z*) to the cylinder’s height direction. The relativistic electron beam generation was simulated using a 2D PIC code in cartesian planar coordinates (*x*, *z*). Finally, 3D hybrid-PIC simulations in (*x*, *y*, *z*) coordinates, calculated the electron propagation inside the target using the parameters of the simulated implosion plasma and the simulated electron beam source as initial conditions. Here, we discuss the results of the hydrodynamic and PIC simulations, which form the basis of the electron transport simulations that will be described in §5.

### (a) Hydrodynamic simulation of the cylindrical target implosion

To describe the implosion of the cylinder target, 2D hydrodynamic simulations were performed using the FLASH code [34,35]. The simulation was made using the experimental parameters of the target and included high-Z foils on each side as well as the protective solid CH tube and cones. An initial external B-field was set to 5 T uniformly along the longitudinal axis. For the EOS and opacity in the FLASH simulation, we used Propaceos tables [36] with a non-LTE model and the opacities are generated using six groups of radiation, distributed logarithmically from 10^−1^ eV to 10^5^ eV.

**Figure 5.**
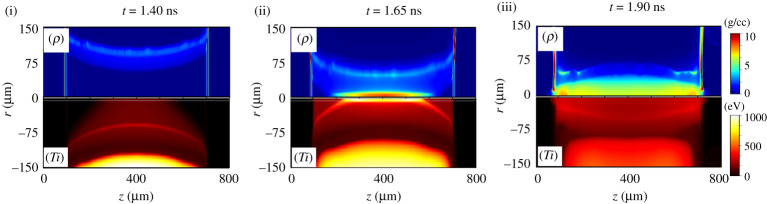
2D distributions of ion mass density and ion temperature at *t* = 1.40 ns, *t* = 1.65 ns and *t* = 1.90 ns. At *t* = 1.40 ns, the temperature increases to 160 eV around the centre of the target due to radiation heating while the compression front has not yet reached the centre. At *t* = 1.65 ns, the temperature at the centre reaches 920 eV. At *t* = 1.90 ns, the temperature decreases to 320 eV due to expansion. (Online version in colour.)

Figure 5 shows the simulation results of the ion density and the ion temperature at *t* = 1.40 ns (i), *t* = 1.65 ns (ii) and *t* = 1.90 ns (iii). At *t* = 1.40 ns, the compression front is still far from the centre of the target and remains in motion. The ion temperature behind the compression front reaches up to 400 eV while the inner plasma, not yet compressed but pre-heated by radiation diffusion, reaches 160 eV all the way to the centre at this time. When the compression front reaches the centre at *t* = 1.65 ns, the ion temperature increases up to 920 eV, and the compressed density reaches 9.0 g cm^−3^. We defined this time as the maximum temperature time. The curvature of the compression front is due to the nonuniform laser irradiation inducing a slower compression front velocity near the cylinder ends. The compressed region is enclosed by 200 μm ≤ *z* ≤ 600 μm, 0 μm ≤ *r* ≤ 20 μm. After *t* = 1.65 ns, the middle of the shock rebounds while the compression reaches the centre closer and closer to the ends. The local temperature gradually decreases through thermal diffusion in the compressed high-density region and then the cooling proceeds due to the expansion after the shock re-bound at the centre. At each time, the electron temperature *T*_*e*_ is almost similar to the ion temperature *T*_*i*_. More details about the implosion dynamics using the same targets and irradiation, without the external B-field, have been reported using FLASH simulations and have been compared with experimental data [32].

**Figure 6.**
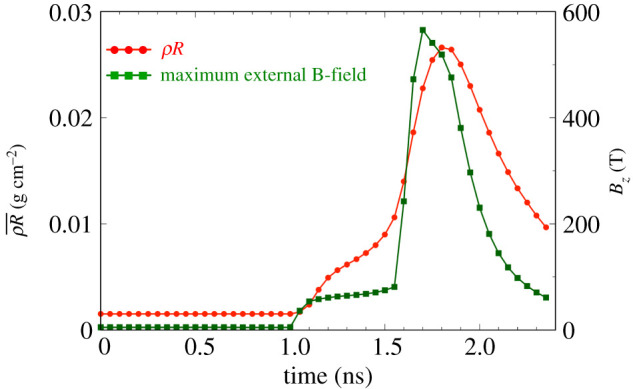
Evolution of $\bar{\rho R}$, which is averaged in the whole CH foam region (100 μm ≤ *z* ≤ 700 μm), and the maximum *B*_*z*_ in the simulation domain. The $\bar{\rho R}$ reaches the maximum value, 0.028 g cm^−2^, at *t* = 1.80 ns while the compressed *B*_*z*_ reaches the maximum value, 580 T, at *t* = 1.70 ns. (Online version in colour.)

The evolution of $\bar{\rho R}$, which is averaged in the whole CH foam region (100 μm ≤ *z* ≤ 700 μm), and the maximum B-field *B*_*z*_ in the simulation domain are shown in figure 6. We can see that the compressed density starts to increase at *t* = 1.05 ns. The $\bar{\rho R}$ and the maximum *B*_*z*_ suddenly increase at a higher rate at *t* = 1.55 ns, indicating that the compression reaches the centre at this time. The maximum *B*_*z*_ peaks at 580 T at *t* = 1.80 ns which is 116 times the initial B-field. Then, the compressed $\bar{\rho R}$ reaches up to 0.028 g cm^−2^ at *t* = 1.90 ns. After that, the density and B-field gradually decrease as the plasma rebounds. Through the implosion process, the beta value (ratio between the plasma pressure and the magnetic pressure) is around 10, which is high enough to consider that the hydrodynamic pressure is dominant. In addition, the Hall parameter (ratio between Larmor frequency and electron–ion collision frequency) is *ω*_*ce*_/*ν*_*ei*_ = 0.076 ≪ 1, where the parameters are taken from the FLASH simulation at *t* = 1.65 ns, that is the time of maximum temperature. Therefore, the magnetic effect is negligible for the implosion dynamics for a 5 T initial field strength. This is also confirmed by the indistinguishable difference of timing between the FLASH simulation with $B_{\text{seed}}=5\;\text{T}$ (figure 5) and the FLASH simulation without external B-field reported in [32].

To compare the simulation with the experimental result at *t* = 1.21 ns, we identified the corresponding timing when the maximum temperature is reached in the FLASH simulation, which is at *t* = 1.65 ns. Still to be understood is the difference of the compression time between the simulation (1.65 ns) and the experiment (1.21 ns), which, as stated before, is unlikely to be caused by the external B-field. We hypothesize that this 0.45 ns shift in timing may arise from: (i) the uncertainties in determining the delays from spectroscopy and/or (ii) other effects inherent to the alignment of the cylindrical target, which may also modify the compression topology and the signal collected by the spectrometer. At this stage, the reason for this delay remains unclear and warrants the acquisition of new experimental data.

### (b) Particle in cell simulation of the intense laser for a parameterized electron source

To determine the source characteristics of electrons accelerated in the interaction with the relativistic high-intensity laser, OMEGA-EP, a kinetic simulation model is needed. We used the fully relativistic 2D PIC code, EPIC [37,38], to simulate the interaction of the OMEGA-EP beam with the high-Z foils located at one end of the cylinder. This code includes ionization processes using a Monte–Carlo scheme computing the cross-section for each process, that is, the ADK model for field ionization [39] and the BEB model for electron impact ionization [40]. The electron impact ionization process is incorporated for electron–electron, ion–ion and electron–ion binary collisions. This approach can reproduce transient plasma dynamics and fast electron generation self-consistently.

The simulation box size is *L*_*x*_ × *L*_*z*_ = 240 μm × 100 μm with a mesh size: Δ*x* = Δ*z* = 0.04 μm( = 0.4 *c*Δ*t*). The simulation boundary conditions for the x- and the z-directions are set as periodic and open for both fields and particles, respectively. The simulation model includes the Al (Z = 13) foil with 8 μm thickness and the Zn (Z = 30) foil behind it, with 10 μm thickness. The initial atom densities in the foil regions are based on solid states, i.e. *ρ*_(Al)_ = 2.7 g cm^−3^ and *ρ*_(Zn)_ = 7.1 g cm^−3^. An Al pre-plasma is depicted as an exponentially decreasing density profile with a length-scale of 1.0 μm. This representation is appropriate according to the hydrodynamic simulations until *t* = 2.0 ns, where the CH plasma expands and mixes with the high-Z foils. The Al foil is set to 32 μm ≤ *z* < 40 μm, and the Al pre-plasma is set to 26 μm ≤ *z* < 32 μm. The Zn foil is set to 40 μm ≤ *z* < 50 μm. The front vacuum extent is 26 μm. To mimic the electron re-circulation from the sheath field, which results from the large charge density gap between the Zn foil and the CH foam, the simulation assumes 50 μm of vacuum at the rear side of the Zn foil instead of the CH foam. The ion macro-particle numbers of Al and Zn foils are 100/mesh and 110/mesh with the same particle weight, respectively. The electron macro-particle numbers depend on the charge state of the ion, i.e. *Z* × 100/mesh and *Z* × 110/mesh. The short pulse high intensity laser assuming OMEGA EP irradiated the Al foil with a peak intensity of $I_{0}=1.1\times 10^{19}\;{\text{W cm}}^{-2}$, using a Gaussian pulse duration of 10 ps (FWHM) and a reduced spot size 13.5 μm diameter for the 80% laser energy (8.86 μm diameter in FWHM) to reduce the electron reflection from the transverse boundary. In this simulation, the external 5 T B-field is neglected since the electron Larmor radius for the relativistic energy is much larger than the cylinder radius.

**Figure 7.**
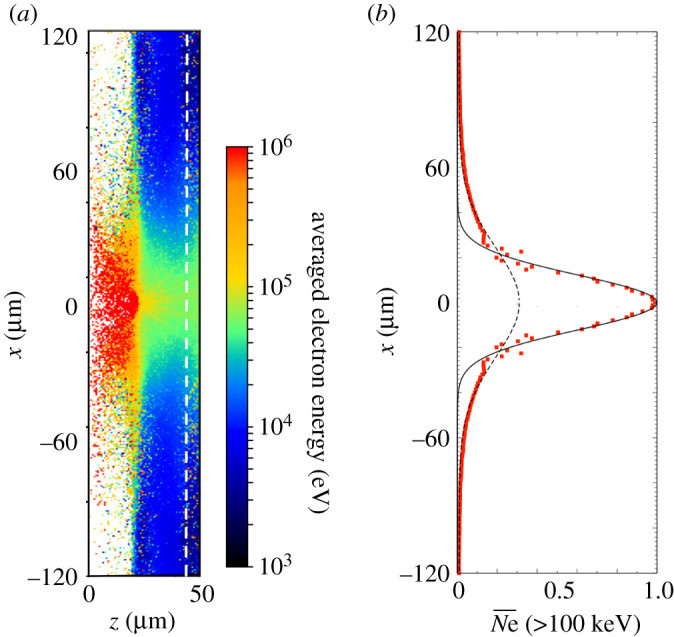
From the 2D PIC simulation of the electron acceleration, a map of the electron energy averaged in each mesh (*a*), and the cumulative spatial distribution of electrons recorded passing through the midplane of the Zn foil as shown by the vertical dashed line (*b*). $\bar{N_{e}}$ is the normalized electron number with the energy greater than 100 keV at each position. The width of the electron profile at this position corresponds to three times the laser spot area. (Online version in colour.)

The fast electrons are mainly accelerated from the Al foil surface. Figure 7 shows a map of the averaged electron energy (figure 7*a*), and the electron spatial distribution measured at the midplane of the Zn foil for electrons above 100 keV (figure 7*b*). At the laser peak irradiation time, the Al and Zn ions near the laser spot area are ionized to the highest charge state of the L-shell. While the field ionization predominantly takes place in the pre-plasma region due to the direct interaction with OMEGA-EP, most of the ions are ionized by the electron impact through non-local electron transport inside the foils. The averaged electron energy reaches 100 keV around the laser spot area, as seen in figure 7*a*. This distribution also shows a filament structure around the centre of the foil target (−10 μm ≤ *x* < 10 μm, 34 μm ≤ *z* < 44 μm), corresponding to the resistive Weibel instability [41]. The diffusion of the accelerated electrons proceeds along the transverse direction through the collisional relaxation process with the bulk plasma. At this time, the spatial distribution of the normalized electron number with the energy greater than 10 0 keV expand laterally to −15 μm < *x* < 15 μm which is a wider region than the laser spot area. At the measurement plane in the Zn foil, the electrons with an energy above 100 keV exhibit a Gaussian profile, as seen in figure 7*b*. The radius of the electron distribution measured at a half depth of the Zn foil corresponds to three times the laser spot radius, *r*_*L*_ = 4.43 μm, as discussed also in [42]. Thus, the electron mean divergence half-angle Θ is estimated from the electron source width after expanding from the laser spot radius and the thickness of the foils (*l*_Al_ = 8.0 μm and *l*_Zn_ = 10.0 μm): $\Theta =\arctan[(3r_{L}-r_{L})/(l_{\text{Al}}+l_{\text{Zn}}/2)]≃34^{\circ }$ with respect to the normal direction. The electron dispersion angle is Δ*θ*_0_ = 55° estimated from the ratio of the averaged electron momentum, $\text{arctan}(p_{x}/p_{z})$.

**Figure 8.**
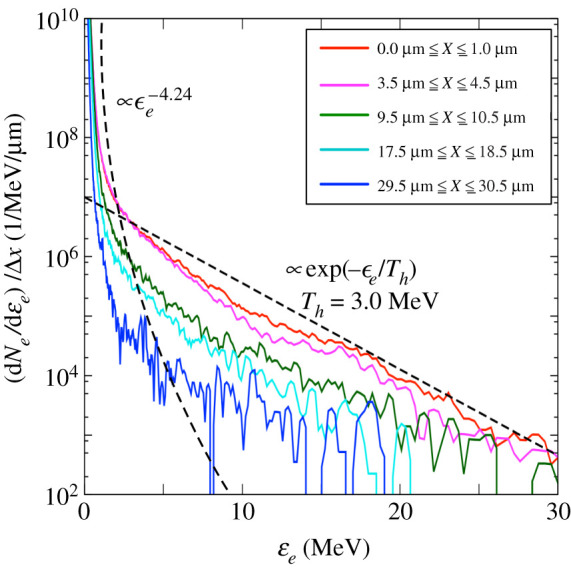
Injected electron spectra counted at the midplane of the Zn foil for each transverse position within Δ*x* = 1.0 μm. The spectra follow the power-low distribution, $\epsilon_{e}^{-4.24}$, and the Maxwell distribution with the slope temperature, *T*_*h*_ = 3.0 MeV. (Online version in colour.)

Figure 8 shows the electron spectra measured at the midplane of the Zn foil for each transverse position with a width of Δ*x* = 1.0 μm. Only the electrons with energy above 100 keV and positive *v*_*z*_ have been included. Note that, to avoid over-counting, the energy recorded for any unique macro particle is overwritten in the case of multiple passes. The electron spectra are accurately parameterized by the following function:

4.1 $$f(\epsilon_{e})=A_{\text{sh}}\epsilon_{e}^{-a}+\exp(-\epsilon_{e}/T_{h})$$

where *A*_sh_ = 10^1^, *a* = 4.24 and *T*_*h*_ = 3.0 MeV. For the high-energy part, the electron spectra are described by the Maxwellian function, which is commonly used to estimate the accelerated electron energy. In figure 8, the hot electron temperatures at the different positions are ranging from 2.51 MeV to 3.34 MeV, which is higher than the ponderomotive scaling (*T*_*p*_ ≃ 1.0 MeV). Here, we approximate a uniform slope temperature of 3.0 MeV to represent all the regions. The reason for this is the electrons are subject to ‘super ponderomotive’ acceleration mechanisms that arise during the multi-picosecond pulse duration, as reported in a few papers [43,44]. The power-law function more accurately describes the spectra for the low-energy part than the Maxwellian function [25,45]. The amount of the measured electrons decreases with the distance from the laser focal spot area, as illustrated by the different electron distributions in figure 8. Yet, the slopes of each electron distribution, which are directly related to the temperature, are almost constant. Thus, the injected electron source approximately conserves the same temperature regardless of the distance, which allows estimating its size, seen in figure 7*b*, The local dispersion angle can be related to the transverse electron temperature, while the local electron propagation angle is related to the beam transverse velocity. In the PIC-hybrid simulations, the beam angular distribution [14,42] is given by

4.2 $$f_{h}(\theta ,r)=\exp\left(-\frac{{\left(\theta -\theta_{r}\right)}^{2}}{\Delta \theta_{0}^{2}}\right),$$

where the dispersion angle Δ*θ*_0_ = 55° and the propagation angle $\theta_{r}=\arctan(\tan(\Theta )r/r_{0})$, with Θ = 34° the mean divergence half-angle and *r*_0_ the beam radius. This form for the angular distribution takes into account the variation of divergence angle with radius, and the parameters Θ, Δ*θ*_0_ are evaluated from the EPIC simulation, averaged over the laser pulse duration. From the EPIC simulation, we estimated the laser absorption rate to be approximately 30 %. For the hybrid simulation, the distributions of the injected electrons are approximated to be the same functions throughout the heater beam interaction.

## 5. Hybrid-PIC simulations of fast electron transport

The fast electron transport and the energy deposition were computed by 3D simulation using the hybrid code developed by Honrubia *et al.* [46]. This code describes the bulk plasma as a fluid model and the fast electron transport with a kinetic model. The included classical Drude model describes the electron resistivity based on the collision frequency given by the Eidmann Chimier model [47,48], which includes the effect of local and non-local collisions.

Each hybrid PIC simulation in this series is initialized with the parameters from a snapshot of the implosion plasma from the FLASH simulation and the injected electron source determined by the EPIC simulations. The plasma density, temperature and compressed external B-field are extracted from the 2D Hydrodynamic simulation for each delay time, as discussed in Sec. III. The 3D parameters are distributed according to axisymmetric rotation of the 2D results. The energy and angular distributions of the injected fast electrons are, respectively, given by the functions of equation (4.1) and (4.2), using the parameters discussed in the previous section. The injection axis is colinear with the cylinder axis. The hybrid simulation considered a 150 μm radius region of interest, with the box size *L*_*x*_ = *L*_*y*_ = 300 μm and *L*_*z*_ = 800 μm. We recall that before the implosion (*τ* = 0 ns), the CH foam is located at 100 μm ≤ *z* ≤ 700 μm, and the high-Z films are located at 90 μm ≤ *z* < 100 μm and 700 μm < *z* ≤ 710 μm. The beam injection position is at *z* = 95 μm. The spatial mesh size is 1 μm in the longitudinal direction (*z*) and 2 μm in the transverse directions (*x* and *y*). We assumed a Gaussian profile for the electron source with the duration of *t* = 10 ps (FWHM), starting 10 ps before the laser peak time, and the total simulation time is 20 ps. The evolution of the absolute number of electrons follows the laser energy profile, multiplied by the absorption rate, i.e. ∝0.3 *I*(*t*, *x*, *y*). As inferred from the PIC simulation, the electron beam initial radius is three times larger than the laser spot radius of the OMEGA-EP laser, i.e. *r*_0_ = 35 μm (HWHM).

### (a) Comparison between simulated electron spectra and the experimental results

**Figure 9.**
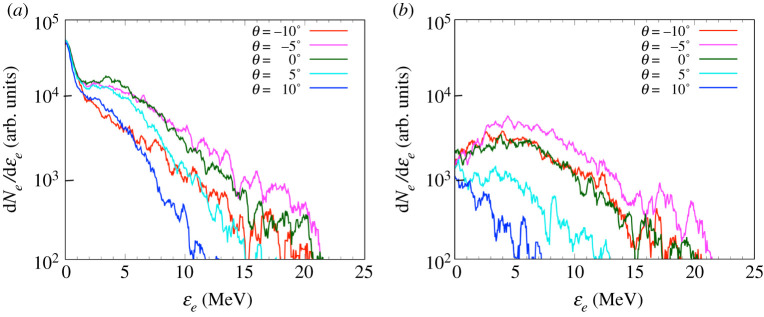
Simulated electron spectra measured at 100 μm away from the rear surface of the cylinder (right-hand side of the simulation domain) for time delays for *τ* = 0.00 ns (*a*) and *τ* = 1.65 ns (*b*). *N*_*e*_ is the normalized electron number, and *ϵ*_*e*_ is the electron kinetic energy. The quantity of electrons at −5° is the highest value among all angles for both time delays. The numbers of electrons gradually decrease for energies less than 5 MeV at *τ* = 1.65 ns. Although the peak of the electron energy distribution shifted from 10 MeV to 5 MeV, the trend is similar to the experimental electron spectra at *τ* = 1.21 ns (figure 4). (Online version in colour.)

The multi-stage simulations, as seen in figure 9*a*,*b*, agree with the electron transport tendencies observed in the experimental results in figure 4*a*,*b* in two ways: (i) there is an asymmetry of the electron beam angular distribution, consistent with the offset position of the EP laser and (ii) there is an increasing reduction of the number of electrons for <10 MeV, which seems consistent with an increased absorption of electrons and the magnetic mirror effect identified in the electron transport simulations. Note that in the hybrid-PIC simulations described in this subsection, the electron source was offset 100 μm away from the centre of the Zn foil and 30° with respect to the OUESM measurement plane, corresponding to the offset observed in the experiment as discussed in §3. For *τ* = 0.00 ns, the number of low-energy electrons in the simulation is higher than in the experiment. It is due to the sheath field excited on the rear surface that reflects more electrons to the backward direction since the detector position in the experiment is located further from the cylinder target than in the simulation. The magnetic mirror effects (resulting mainly from the closing cone structure of self-generated resistive *B*_*y*_, discussed in details in the next section) occur near the maximum compression time, which is consistent with the gradual decrease of the electron numbers for energies less than 10 MeV at *τ* = 1.65 ns. These results show similar features to the experimental results at *τ* = 1.21 ns, although the peak of the electron energy distribution shifted from 10 MeV in the experiment to 5 MeV in the simulations. The number of transmitted electrons also presents an asymmetry in angular distribution, due to the asymmetry between the cylinder foam structure and the injected electron position introduced by the beam offset. With the offset, the quantity of electrons at −5° is the highest value among all angles. It decreases with the increase of the angle, as observed from the angular dependence of the experimental results.

### (b) Electron transport mechanism considering OMEGA-EP irradiation along the centre axis of the cylinder target

**Figure 10.**
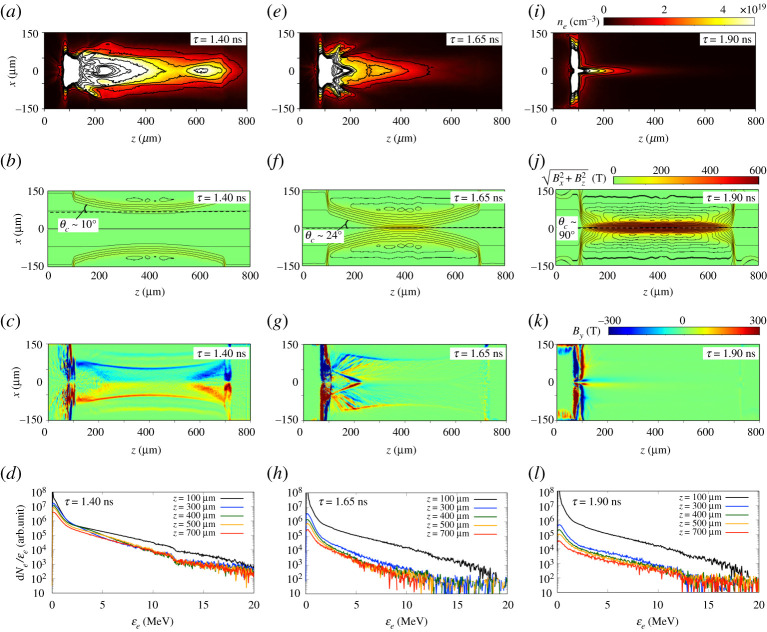
Distributions at 10 ps from the simulation starts (slice at *y* = 0) of fast electron density, external $\sqrt{B_{x}^{2}+B_{z}^{2}}$, and self-generated *B*_*y*_ in the *x*–*z* plane for time delays *τ* = 1.40 ns (*a*–*c*), *τ* = 1.65 ns (*d*–*f* ) and *τ* = 1.90 ns (*g*–*i*). The closing cone angles *θ*_*c*_ near the injection plane are represented in (*b*,*e*,*h*). The contours in (*b*,*e*,*h*) show constant magnetic potential, which correspond to the B-field lines. The dashed line shows the position of the compression front. Electron spectra extracted at the different positions along the cylinder (*z* = 100 μm, 300 μm, 400 μm, 500 μm and 700 μm), integrated in the region of *r* < ≤20 μm for the time delays *τ* = 1.40 ns (*d*), 1.65 ns (*h*) and 1.90 ns (*l*). (Online version in colour.)

In the hybrid-PIC simulation results that follow, the electron source was aligned to the centre axis of the cylinder target (disregarding the OMEGA EP offset) in order to conduct a systematic study of the electron transport phenomena and energy deposition efficacy that can be expected in a magnetized cylindrical implosion. Simulations with the same source are carried out at each time delay. Figure 10 shows the simulation results at 10 ps from the simulation starts (slice at *y*=0) of the fast electron density, the external $\sqrt{B_{x}^{2}+B_{z}^{2}}$, and the self-generated *B*_*y*_ at *τ* = 1.40 ns (*a*–*c*), *τ* = 1.65 ns (*e*–*g*) and *τ* = 1.90 ns (*i*–*k*). In addition, the electron spectra extracted at the different positions along the cylinder, integrated in the region of *r* ≤ 20 μm are shown in figure 10*d*,*h*,*l* at the time delays *τ* = 1.40 ns, 1.65 ns and 1.90 ns, respectively. For *τ* = 1.40 ns, the electrons are concentrated near the front surface (*z* = 250 μm) and near the rear surface (*z* = 650 μm) as seen in figure 10*a*. At this delay, the compressed external $\sqrt{B_{x}^{2}+B_{z}^{2}}$ is still far from the centre axis, as seen in figure 10*b*. However, the inner self-generated *B*_*y*_ at the radiation heated front and the outer self-generated *B*_*y*_ at the compression front (figure 10*c*) pinch the electron beam at the cylinder edges. For *τ* = 1.65 ns, when the compression reaches the centre in figure 10*f* , the self-generated *B*_*y*_ converges to the centre axis and forms a closing cone structure near the injection plane, with the corresponding angle *θ*_*c*_ ∼ 24° shown in figure 10*g*. Its effects on the transport of electrons will be detailed below. For *τ* = 1.90 ns, when the compression reaches the cylinder ends as seen in figure 10*j*, most of the injected electrons are trapped in the Zn foil region; only a minuscule fraction of the high-energy electrons are collimated along the compressed B-field and propagate to the inside, as seen in figure 10*i*.

The main magnetic mirror that is detrimental to the transport of electrons inside the foam for *τ* = 1.65 ns and *τ* = 1.90 ns is due to the cone structure of the self-generated *B*_*y*_. Let us consider the case for which the electrons have a smaller Larmor radius than the width of the self-generated *B*_*y*_ structure. The electron reflection can be estimated by the injected electron mean divergence half-angle *θ*_*inj*_ = Θ ( = 34°) calculated by the PIC simulation and the declined cone angles *θ*_*c*_ ≃ 10°, 24° and 90° for *τ* =1.40 ns, 1.65 ns and 1.90 ns, as displayed in (figure 10*b*,*f* , *j*). Through the electron synchrotron motion by the self-generated *B*_*y*_, the reflection angle to the cylinder axis is given by $\theta_{\text{r}}=\theta_{\text{inj}}+2n\theta_{\text{c}}$, where *n* is the number of reflections in the cone. In order for electrons to move forward, the condition of $\theta_{\text{inj}}+2n\theta_{\text{c}}<90^{\circ }$ is necessary to be satisfied until the electrons reach the cone edge. At *τ* = 1.40 ns, when the compression front has not yet reached the centre, a large number of the injected electrons with the density of *n*_*b*_ ≤ 10^19^ cm^−3^ goes through the inner self-generated *B*_*y*_, which is excited at the front pre-heated by radiation diffusion and that is lower than 100 T. The outer self-generated *B*_*y*_, which is excited at the compression front of the implosion, reaching over 200 T confines those electrons to the inside of the target due to the flat compression surface with $\theta_{\text{c}}=10^{\circ }$. The propagating electrons are re-focused near the rear surface through these processes. For *τ* = 1.65 ns, the amplitude of the self-generated *B*_*y*_ excited within the closing structure at 100 μm ≤ *z* ≤ 200 μm and *r* < 100 μm reaches around 300 T. Electrons with energies less than 2 MeV (Larmor radius: *r*_*L*_ < 2.7 μm) are thus reflected within the ≃10 μm stroke width of the self-generated *B*_*y*_ structure. Most of the electrons with mean injection angle $\theta_{\text{inj}}=34^{\circ }$ are reflected at their second reflection (*n* = 2) for the cone angle $\theta_{\text{c}}=24^{\circ }$, leading to an effective propagation distance of only ∼54 μm. For *τ* = 1.90 ns, the amplitude of the self-generated *B*_*y*_ increases along the Zn foil surface, and the cone angle drastically increases to $\theta_{\text{c}}≃90^{\circ }$. Therefore, only a few electrons with a Larmor radius larger than the self-generated *B*_*y*_ width escape from the self-generated *B*_*y*_ region. Moreover, the magnetic mirror effect of the compressed external $\sqrt{B_{x}^{2}+B_{z}^{2}}$ is also suspected to take place when the maximum external magnetic amplitude reaches *B*_*z*_ = 580 T around 1.70 ns on the cylinder axis, which corresponds to 116 times the initial amplitude of the external $\sqrt{B_{x}^{2}+B_{z}^{2}}$, $B_{\text{seed}}=5\;\text{T}$. The maximum angle to pass through the mirror is $\theta_{\text{max}}=5.3^{\circ }$, estimated by the relation of $B_{\text{seed}}/B_{\text{z}}=\sin^{2}\theta_{\text{max}}$. However, since the self-generated B-field reflects most of the electrons before reaching the compressed region, the mean angle of electrons that had enough energy to pass through the first mirror and get to *z* > 200 μm is smaller than 5.3°. Hence, these high-energy electrons can penetrate deeper in the foam and are instead guided by the external $\sqrt{B_{x}^{2}+B_{z}^{2}}$ field.

The electron reflection for each time delay is seen in the electron spectra extracted at the different positions in figure 10*d*,*h*,*l*. At *τ* = 1.40 ns, most of the electrons are injected into the CH foam region. The self-generated B-field structure is located away from the centre, thereby allowing for the electrons to be injected in the foam without the mirror effect. Note that the reduction of their number is due to energy deposition in the foam and electrons leaving the region of *r* ≤ 20 μm. At *τ* = 1.65 ns and *τ* = 1.90 ns, a significant number of the injected electrons are reflected at the front by the mirror effect of the self-generated B-field. In addition, the number of electrons with energy *ϵ*_*e*_ > 3 MeV at *z* ≥ 400 μm remains approximately the same, while the electron number with *ϵ*_*e*_ ≤ 3 MeV decreases with the propagation and absorption into the CH foam. At *τ* = 1.90 ns, the energy range contributing to the heating of CH foam extends to around 5 MeV, with the increase of the compressed density.

**Figure 11.**
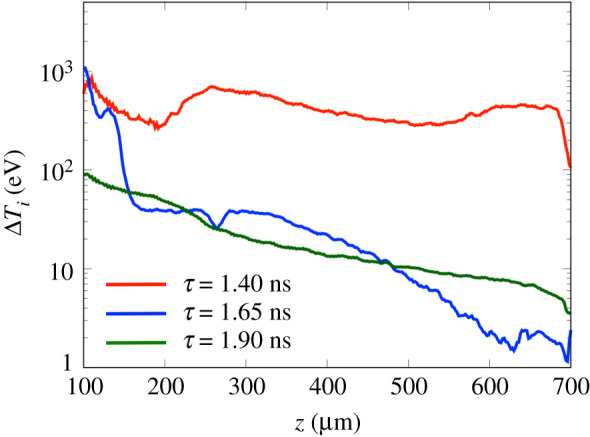
Increased ion temperature profiles along the cylindrical centre axis, defined by the difference of the temperature during 20 ps with Δ*T*_*i*_ = *T*_*i*_(*τ* + 20 ps) − *T*_*i*_(*τ*) from the electron injection for each time delay (*τ* = 1.40 ns, *τ* = 1.65 ns and *τ* = 1.90 ns). (Online version in colour.)

Figure 11 shows the increase of the ion temperature, Δ*T*_*i*_(*z*, 0), due to the electron transport along the cylindrical centre axis for each time delay (*τ* = 1.40 ns, *τ* = 1.65 ns and *τ* = 1.90 ns), where Δ*T*_*i*_(*z*, *r*) is defined as the difference between the final and initial temperature during the 20 ps following electron injection, i.e. Δ*T*_*i*_ = *T*_*i*_(*τ* + 20 ps) − *T*_*i*_(*τ*). For *τ* = 1.40 ns, the increase of the ion temperature Δ*T*_*i*_ is >520 eV around *z* = 250 μm and *z* = 650 μm, corresponding to the electron focusing positions discussed above. Δ*T*_*i*_ at the half of the cylinder length (*z* = 400 μm) reaches up to 400 eV in the initial CH density of 0.1 g cm^−3^. For *τ* = 1.65 ns, Δ*T*_*i*_ starts to decrease from *z* = 150 μm which roughly corresponds to the propagation limit estimated by the self-generated B-field reflection. After this region, Δ*T*_*i*_ becomes a constant value of 40 eV. From the compressed region, *z* = 350 μm, Δ*T*_*i*_ decreases due to the magnetic mirror effect of the external B-field. For *τ* = 1.90 ns, Δ*T*_*i*_ exponentially decreases because of the energy deposition. At this time Δ*T*_*i*_ is 10 eV at (*z* = 400 μm), which is 40 times less than at *τ* = 1.40 ns. However, since the ion mass density is 80 times greater than the pre-compressed density, the energy deposited around the cylinder axis due to fast electrons peaks again after the compression.

**Figure 12.**
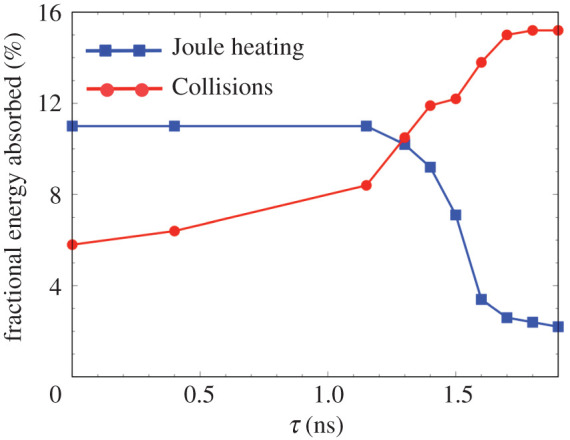
Fractional energy (normalized by the injected electron energy) absorbed in the whole target, including the high-Z foils on both edge, for the time delay of *τ*. The red line shows the direct absorption through collisions between the bulk plasma and the injected electrons (collisional heating), and the blue line showsthe indirect absorption through the collision between the bulk plasma and the return current excited by the electron beam (Joule heating). The collisional heating is dominant after the compression while the Joule heating mainly takes place before the compression. (Online version in colour.)

The heating due to the fast electrons occurs through two distinct processes: Joule heating by the high-energy component and collisional heating by the low-energy component. The Joule heating, which is mediated through a return current of electrons, is most observable before the compression time, while the collisional heating due to direct binary collisions occurs after. Figure 12 plots the fractional energy (normalized by the injected electron energy) absorbed in the whole target, including the high-Z foils on both edge, through Joule heating and collisions for each electron transport simulated time delay. For *τ* ≤ 1.40 ns, the energy indirectly absorbed by Joule heating is almost constant, corresponding to 11% of the injected electron energy. At that time, the high current density component of the electron beam propagates into the inside of the CH foam. However, this component suddenly decreases after *τ* = 1.40 ns when the high-density plasma of the compression closes to the electron injection region. The collisional effect then dominates and increases as the implosion proceeds. For *τ* = 1.60 ns, the absorbed energy due to collisions reaches up to 13.8%, while that of the Joule heating decreases to 2.2%. However, after the compression, most of the electrons are reflected at the front and also heat the Zn foil through the collisional process. The heating at the Zn foil corresponds to almost half of the total collisional heating.

**Figure 13.**
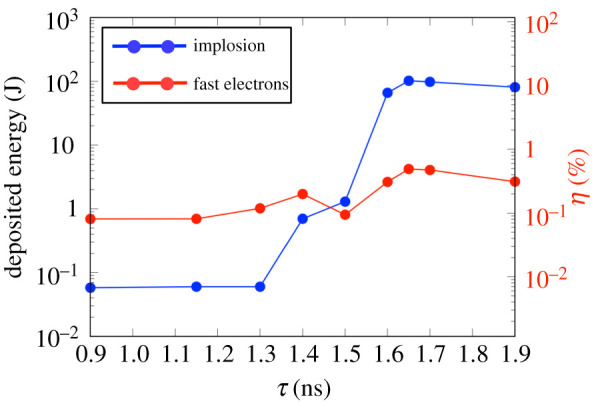
Deposited energy integrated in the region 200 μm ≤ *z* ≤ 600 μm, $0\leq r=(\sqrt{x^{2}+y^{2}})\leq 20\;\mu \text{m}$ (corresponding to the compressed region at *τ* = 1.65 ns). The curves show the energy integrated in this region at the implosion time *t* = *τ* (blue) and the additional energydue to the fast electrons at *t* = *τ* + 20 ps (red). The conversion efficiency *η* from the irradiated OMEGA-EP laser energy into this compressed region is shown in the right axis. The energy deposited by the implosion reaches 118.2 J at *τ* = 1.65 ns. The energy due to fast electrons has two peaks of 1.2 J and 3.8 J at the time delays of *τ* = 1.4 ns and *τ* = 1.65 ns, respectively. (Online version in colour.)

Figure 13 shows the energies deposited around the centre of the cylinder target at each time delay for which a transport simulation was performed. The energy is estimated from *n*_*i*_[*T*_*i*_(*τ* + 20 ps) − *T*_*i*_(*τ*)] + *n*_*e*_[*T*_*e*_(*τ* + 20 ps) − *T*_*e*_(*τ*)] summed over the integration region (200 μm ≤ *z* ≤ 600 μm, *r* ≤ 20 μm) which is defined by the compressed profile at *t* = 1.65 ns, i.e. 200 μm ≤ *z* ≤ 600 μm, $0\leq r\left(=\sqrt{x^{2}+y^{2}}\right)\leq 20\;\mu \text{m}$. The lines show the energy deposition due only to the implosion and additional heating by the fast electrons. The two processes cause peak energy deposition at two different times: *τ* = 1.40 ns and *τ* = 1.65 ns. Simulations for times earlier than *τ* = 1.40 ns show the optimum in terms of a large number of electrons guided towards the rear side of the cylinder. The additional energy reaches 1.2 J, of which 80% is due to the binary collision and the remaining 20% is due to the Joule heating. The implosion process mainly heats the cylinder on the second peak at *τ* = 1.65 ns. Although the total energy reaches up to 121.5 J, the heating due to the fast electron transport is only 0.49% of the irradiated OMEGA-EP energy. This is due to the self-generated B-field, which reflects most of the fast electrons at the front of the CH foam. After *τ* = 1.65 ns, the total energy decreases with the expansion of the compressed area, while the fast electron heating remains low.

## 6. Predicted improvements with stronger external B-field

**Figure 14.**
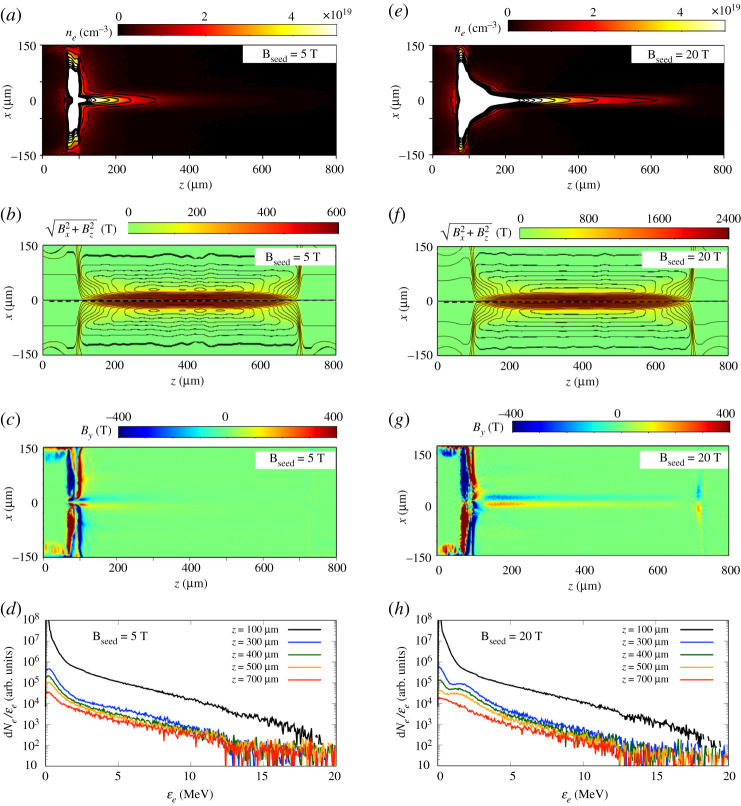
Distributions of fast electron density, external B-field, self-generated B-field, and transmitted electron spectra in the case with $B_{\text{seed}}=5\;\text{T}$ in (*a*–*d*) and 20 T in (*e*–*h*). The outermost black contours in (*a*) and (*e*) show the surface where density *n*_*e*_ = 1.0 × 10^19^ cm^−3^. The contours in (*b*) and (*f* ) show constant magnetic potential, which correspond to the B-field lines. The electron spectra in (*d*) and (*h*) are extracted at the different positions along the cylinder (*z* = 100 μm, 300 μm, 400 μm, 500 μm and 700 μm), integrated in the region of *r* ≤ 20 μm. (Online version in colour.)

After the shock front reaches the centre of the cylinder target, the compressed external B-field becomes strong enough to guide the fast electrons in the forward direction. In order to quantify the dependence on the external B-field strength for the high-energy electron heating, we carried out additional FLASH simulations with *B*_seed_ = 10, 20, 30 and 40 T and the associated hybrid PIC simulations. Figure 14 shows the simulation results (slice at *y* = 0) of the fast electron density, the external $\sqrt{B_{x}^{2}+B_{z}^{2}}$, the self-generated *B*_*y*_ and the electron spectra at different z positions for the time delay *τ* = 1.90 ns with *B*_seed_ = 5 T and 20 T. In figure 14*a*,*e*, one can see that the injected electrons can penetrate to the inside of the foam in the case of higher external $\sqrt{B_{x}^{2}+B_{z}^{2}}$. The external $\sqrt{B_{x}^{2}+B_{z}^{2}}$ in the *B*_seed_=20 T case of figure 14*f* is compressed to a maximum value of *B*_*z*_ ≃ 2300 T at *τ* = 1.90 ns. The compressed *B*_*z*_ near the front surface in figure 14*f* becomes larger than the self-generated *B*_*y*_ in figure 14*g*. Consequently, the injected electron density profile in figure 14*e* shows that a larger number of electrons were guided by the compressed *B*_*z*_ and could penetrate ahead of the self-generated B-field mirror located at *z* = 100 μm. In the region of 100 μm ≤ *z* ≤ 200 μm the compressed external $\sqrt{B_{x}^{2}+B_{z}^{2}}$ also exhibits a closing structure away from the centre, as seen in figure 14*f* . It induces a magnetic mirror for electrons away from the centre, similar to what has been described previously for *B*_seed_ = 5 T at the same time. Therefore, the guiding is still most effective for electrons located near the axis when they reach *z* = 100 μm, but it is the case of a higher number of electrons for a higher seed B-field, as explained above. Correlated to the increase of the transmitted electrons to the inside of the foam, the self-generated *B*_*y*_ around the central axis becomes larger than in the case with seed B-field of 5 T. This self-generated *B*_*y*_ further confines the electrons to the central axis. Comparing the electron spectra of figure 14*d*,*h*, the electrons with an energy around 2 MeV in the 20 T seed B-field case increase compared with the electron spectrum of the 5 T case, showing the guiding enhancement with higher seed B-field. This electron number with an energy around 2 MeV decreases with the propagation, and the absorbed energy heats the compressed cylinder. In conclusion, the magnetic mirror caused by the self-generated *B*_*y*_ remains similar when increasing $B_{\text{seed}}$, while the external compressed magnetic field directly scales with $B_{\text{seed}}$. Hence, by increasing the seed B-field value for a similar implosion, the external compressed B-field becomes stronger than the self-generated *B*_*y*_ near the injection and electrons can propagate deeper into the foam, eventually guided along the cylinder axis by the combination of the compressed external $\sqrt{B_{x}^{2}+B_{z}^{2}}$ and the azimuthal self-generated *B*_*y*_.

**Figure 15.**
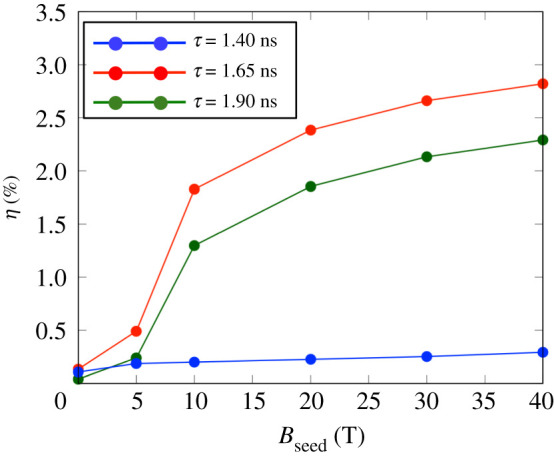
Conversion efficiency *η* from the irradiated OMEGA-EP laser energy into the compressed plasma (200 μm ≤ *z* ≤ 600 μm, $0\leq r\left(=\sqrt{x^{2}+y^{2}}\right)\leq 20\;\mu \text{m}$) for the external seed B-field strength. The lines show the efficiency for the different time delay, *τ* = 1.40 ns, *τ* = 1.65 ns and *τ* = 1.90 ns. The efficiency before the compression, *τ* = 1.40 ns, is less than *η* = 0.20% in $B_{\text{seed}}=40\;\text{T}$. The efficiency after the compression reaches to the centre, *τ* = 1.65 ns, and *τ* = 1.90 ns, becomes *η* ≥ 1.0% from $B_{\text{seed}}=10\;\text{T}$. (Online version in colour.)

The conversion efficiencies *η* from the irradiated OMEGA-EP laser energy into the cylinder plasma energy respect to the initial strength of the external B-field ($B_{\text{seed}}=0-40\;\text{T}$) are plotted for the three-timing of the OMEGA-EP irradiation, *τ* = 1.40 ns, 1.65 ns and 1.90 ns, in figure 15. The energy deposited by the fast electrons is summed over the same compressed region used for figure 13. Before the compression time, *τ* = 1.40 ns, for the case with $B_{\text{seed}}=5\;\text{T}$, the conversion efficiency *η* is slightly larger than that without the external B-field. After this time, *τ* = 1.65 ns and *τ* = 1.90 ns, there are clear differences of *η* between simulations with and without external B-field. In the case without the external B-field, *η* is only 0.04% at *τ* = 1.90 ns since the reflection of the self-generated B-field is dominant. However, in the case with 5 T, a part of the high-energy electrons reaches the centre and heats the compressed target at a certain level. The energy deposition rate reaches around *η*_*e*_ = 0.23%. It is a small value compared with that of the implosion heating. However, it indicates that the external B-field in the experiment was sufficient to play a guiding role in fast electron transport into the compressed cylinder. The B-field guiding primarily depends on the initial strength of the external B-field. In the case of $B_{\text{seed}}=10\;\text{T}$, the conversion efficiencies near the maximum compression time, *τ* = 1.65 ns and *τ* = 1.90 ns, significantly increase to *η* = 1.80% and *η* = 1.27%, respectively, as the compressed B-field becomes larger than the self-generated B-field near the front surface of the cylinder target. For a stronger external B-field, *B*_seed_ > 10 T, the efficiencies at *τ* = 1.65 ns and *τ* = 1.90 ns keep increasing with $B_{\text{seed}}$, while the efficiency at *τ* = 1.40 ns is almost constant around *η* = 0.20%. In the case of $B_{\text{seed}}=40\;\text{T}$, it reaches up to *η* = 2.77% at the delay of *τ* = 1.65 ns.

## 7. Conclusion

We carried out experiments and simulations to understand fast electron transport in order to control energy deposition into a previously characterized, imploded cylindrical CH plasma magnetized with an external B-field. In our approach, the compression of the cylinder target inside a 5 T seed B-field was performed by 36 beams of the OMEGA laser to achieve high-density and high B-field strength. Then, the OMEGA EP laser (*I*_0_ = 1.1 × 10^19^ W cm^−2^) was used to produce relativistic electrons to heat the imploded cylindrical plasma. Our multi-stage simulation approach, including three types of numerical tools, modelled the physics from implosion to electron transport inside the cylinder. The 2D FLASH simulation showed the implosion dynamics of the cylinder target, estimating the maximum compression ratio of the ion density, the ion temperature and the external B-field at over 90 times their initial values. 2D fully relativistic PIC simulation using the EPIC code estimated the electron spectra which we parameterized with a low-energy power-law function (*ϵ*^−4.24^), a high-energy Maxwellian function (*T*_hot_ = 3.0 MeV) and spatial dependence. That source was injected in a series of 3D PIC-hybrid simulations to identify the fundamental mechanisms of the electron transport dynamics, using the FLASH-simulated density, temperatures, and fields at various time-delays for the initial conditions. Although the simulations are not exactly reproducing the experimental results, we could identify main tendencies impacting the transmitted electron: that is (i) a decrease of the number of low-energy electrons due to the magnetic mirror effect and absorption in the foam, and (ii) a similar angular dependence compared with the electron spectra measured in the experiment when considering the OMEGA-EP beam pointing offset. In addition, the simulations conveyed the presence of strong magnetic mirror effects occurring near the maximum compression time and allowed to elucidate the role of self-generated B-field and compressed external B-field in the electron propagation efficiency. The simulation series identified two critical moments of interest during the implosion for the injection of the fast electrons. First, there is an optimal time for electron guiding due to the self-generated B-field, which increases the ion temperature in the uncompressed region before the compression reaches the centre. Most of the injected electrons are then focused by the inner and the outer self-generated B-field wall and heat the cylinder through Joule heating. The increased ion temperature due to the electron transport reaches up to 520 eV through Joule heating. The second opportune time for electron collimation arises near the maximum compression time when the external B-field guides the electrons into the high-density area. After the maximum compression, the magnetic mirror effect arises from the resistive self-generated B-fields at the front of the compression, collapsing along the cylinder axis and leaving closing structures. The angle of the closing self-generated B-field structure increases with the compression getting closer to the front surface of the cylinder, thereby increasing the mirror ratio that prevents the electron to propagate deep inside the foam. This field then reflects the low-energy electrons backward while a few high-energy electrons can go through the compressed cylinder. Under these conditions, the energy deposition rate normalized by the short pulse laser energy reaches the maximum value of 0.49% through mainly collisional heating. Although the number of the penetrating electrons after the compression decreases due to the magnetic mirror effect, the energy deposition rate at the maximum compression time becomes higher than that before the compression.

Finally, the simulation approach was used to explore modifications to the set up that could address the issues and further enhance the electron energy deposition. It was found that a higher seed B-field can, in turn, create the conditions for a stable guiding channel along the axis and synergistic coupling with the self-generated fields.

## Data accessibility

The supporting data for this paper includes raw data from diagnostics used and it can be found in the electronic supplementary material.

## References

[1] Tabak M, Hammer J, Glinsky ME, Kruer WL, Wilks SC, Woodworth J, Campbell EM, Perry MD, Mason RJ 1994 Ignition and high gain with ultrapowerful lasers. Phys. Plasmas 1, 1626–1634. (10.1063/1.870664)

[2] Wilks SC, Kruer WL, Tabak M, Langdon AB 1992 Absorption of ultra-intense laser pulses. Phys. Rev. Lett. 69, 1383–1386. (10.1103/PhysRevLett.69.1383) 10047203

[3] Haines MG, Wei MS, Beg FN, Stephens RB 2009 Hot-electron temperature and laser-light absorption in fast ignition. Phys. Rev. Lett. 102, 045008 (10.1103/PhysRevLett.102.045008) 19257435

[4] Atzeni S *et al.* 2008 Fast ignitor target studies for the HiPER project. Phys. Plasmas 15, 056311 (10.1063/1.2895447)

[5] Robinson AP, Strozzi DJ, Davies JR, Gremillet L, Honrubia JJ, Johzaki T, Kingham RJ, Sherlock M, Solodov AA 2014 Theory of fast electron transport for fast ignition. Nucl. Fusion 54, 054003 (10.1088/0029-5515/54/5/054003)

[6] Norreys P *et al.* 2014 Fast electron energy transport in solid density and compressed plasma. Nucl. Fusion 54, 054004 (10.1088/0029-5515/54/5/054004)

[7] Bellei C, Divol L, Kemp AJ, Key MH, Larson DJ, Strozzi DJ, Marinak MM, Tabak M, Patel PK 2013 Fast ignition: dependence of the ignition energy on source and target parameters for particle-in-cell-modelled energy and angular distributions of the fast electrons. Phys. Plasmas 20, 052704 (10.1063/1.4804277)

[8] Kodama R *et al.* 2002 Fast heating scalable to laser fusion ignition. Nature 418, 933–934. (10.1038/418933a) 12198536

[9] Azechi H*et al.* 2009 Plasma physics and laser development for the fast-ignition realization experiment (FIREX) project. Nucl. Fusion 49, 104024 (10.1088/0029-5515/49/10/104024)

[10] Shiraga H, Nagatomo H, Theobald W, Solodov AA, Tabak M 2014 Fast ignition integrated experiments and high-gain point design. Nucl. Fusion 54, 054005 (10.1088/0029-5515/54/5/054005)

[11] Theobald W *et al.* 2011 Initial cone-in-shell fast-ignition experiments on OMEGA. Phys. Plasmas 18, 056305 (10.1063/1.3566082)

[12] Jarrott LC *et al.* 2016 Visualizing fast electron energy transport into laser-compressed high-density fast-ignition targets. Nat. Phys. 12, 499–504. (10.1038/nphys3614)

[13] Jarrott LC *et al.* 2017 Transport and spatial energy deposition of relativistic electrons in copper-doped fast ignition plasmas. Phys. Plasmas 24, 102710 (10.1063/1.4999108)

[14] Bailly-Grandvaux M *et al.* 2018 Guiding of relativistic electron beams in dense matter by laser-driven magnetostatic fields. Nat. Commun. 9, 102 (10.1038/s41467-017-02641-7) 29317653 PMC5760627

[15] Strozzi DJ, Tabak M, Larson DJ, Divol L, Kemp AJ, Bellei C, Marinak MM, Key MH 2012 Fast-ignition transport studies: realistic electron source, integrated particle-in-cell and hydrodynamic modeling, imposed magnetic fields. Phys. Plasmas 19, 072711 (10.1063/1.4739294)

[16] Sakata S *et al.* 2018 Magnetized fast isochoric laser heating for efficient creation of ultra-high-energy-density states. Nat. Commun. 9, 3937 (10.1038/s41467-018-06173-6) 30258053 PMC6158241

[17] Matsuo K *et al.* 2020 Petapascal pressure driven by fast isochoric heating with a multipicosecond intense laser pulse. Phys. Rev. Lett. 124, 035001 (10.1103/PhysRevLett.124.035001) 32031862

[18] Nagatomo H *et al.* 2015 Computational study of magnetic field compression by laser-driven implosion. Nucl. Fusion 55, 093028 (10.1088/0029-5515/55/9/093028)

[19] Johzaki T, Taguchi T, Sentoku Y, Sunahara A, Nagatomo H, Sakagami H, Mima K, Fujioka S, Shiraga H 2015 Control of an electron beam using strong magnetic field for efficient core heating in fast ignition. Nucl. Fusion 55, 053022 (10.1088/0029-5515/55/5/053022)

[20] Johzaki T *et al.* 2016 Integrated simulation of magnetic-field-assist fast ignition laser fusion. Plasma Phys. Controlled Fusion 59, 014045 (10.1088/0741-3335/59/1/014045)

[21] Slutz SA, Herrmann MC, Vesey RA, Sefkow AB, Sinars DB, Rovang DC, Peterson KJ, Cuneo ME 2010 Pulsed-power-driven cylindrical liner implosions of laser preheated fuel magnetized with an axial field. Phys. Plasmas 17, 056303 (10.1063/1.3333505)

[22] Davies JR, Barnak DH, Betti R, Campbell EM, Chang PY, Sefkow AB, Peterson KJ, Sinars DB, Weis MR 2017 Laser-driven magnetized liner inertial fusion. Phys. Plasmas 24, 062701 (10.1063/1.4984779)

[23] Sweeney MA, Farnsworth AV 1981 High-gain, low-intensity ICF targets for a charged-particle beam fusion driver. Nucl. Fusion 21, 41–54. (10.1088/0029-5515/21/1/004)

[24] Pérez F *et al.* 2011 Magnetically guided fast electrons in cylindrically compressed matter. Phys. Rev. Lett. 107, 065004 (10.1103/PhysRevLett.107.065004) 21902333

[25] Vauzour B *et al.* 2014 Unraveling resistive versus collisional contributions to relativistic electron beam stopping power in cold-solid and in warm-dense plasmas. Phys. Plasmas 21, 033101 (10.1063/1.4867187)

[26] Vauzour B *et al.* 2011 Laser-driven cylindrical compression of targets for fast electron transport study in warm and dense plasmas. Phys. Plasmas 18, 043108 (10.1063/1.3578346)

[27] Del Sorbo D *et al.* 2015 Approach to the study of fast electron transport in cylindrically imploded targets. Laser Part. Beams 33, 525–534. (10.1017/S0263034615000592)

[28] Macchi A, Borghesi M, Passoni M 2013 Ion acceleration by superintense laser-plasma interaction. Rev. Mod. Phys. 85, 751–793. (10.1103/RevModPhys.85.751)

[29] Pereira NR, Davis J 1988 X rays from z-pinches on relativistic electron-beam generators. J. Appl. Phys. 64, R1–R27. (10.1063/1.341808)

[30] Gong Z, Mackenroth XQYF, Arefiev AV 2019 Radiation reaction as an energy enhancement mechanism for laser-irradiated electrons in a strong plasma magnetic field. Sci. Rep. 9, 17181 (10.1038/s41598-019-53644-x) 31748597 PMC6868192

[31] Perez F *et al.* 2010 Enhanced isochoric heating from fast electrons produced by high-contrast, relativistic-intensity laser pulses. Phys. Rev. Lett. 104, 085001 (10.1103/PhysRevLett.104.085001) 20366940

[32] Doziéres M *et al.* 2020 Characterization of an imploding cylindrical plasma for electron transport studies using x-ray emission spectroscopy. Phys. Plasmas 27, 023302 (10.1063/1.5125271)

[33] Habara H *et al.* 2019 A ten-inch manipulator (TIM) based fast-electron spectrometer with multiple viewing angles (OU-ESM). Rev. Sci. Instrum. 90, 063501 (10.1063/1.5088529) 31255022

[34] Fryxell B *et al.* 2000 FLASH: an adaptive mesh hydrodynamics code for modeling astrophysical thermonuclear flashes. Astrophys. J. Suppl. Ser. 131, 273–334. (10.1086/317361)

[35] Tzeferacos P *et al.* 2015 FLASH MHD simulations of experiments that study shock-generated magnetic fields. High Energy Density Phys. 17, 24–31. 10th International Conference on High Energy Density Laboratory Astrophysics (10.1016/j.hedp.2014.11.003)

[36] MacFarlane JJ, Golovkin IE, Woodruff PR 2006 HELIOS-CR-A 1-D radiation-magnetohydrodynamics code with inline atomic kinetics modeling. J. Quant. Spectrosc. Radiat. Transfer 99, 381–397. Radiative Properties of Hot Dense Matter (10.1016/j.jqsrt.2005.05.031)

[37] Kawahito D, Kishimoto Y 2017 Multi-phase ionization dynamics of carbon thin film irradiated by high power short pulse laser. Phys. Plasmas 24, 103105 (10.1063/1.4986034)

[38] Kawahito D, Kishimoto Y 2020 Ionization and acceleration of multiply charged gold ions in solid film irradiated by high intensity laser. Phys. Plasmas 27, 033108 (10.1063/1.5140493)

[39] Ammosov MV, Delone NB, Kraĭnov VP 1986 Tunnel ionization of complex atoms and of atomic ions in an alternating electromagnetic field. Sov. Phys. JETP 64, 2008–2013.

[40] Kim YK, Rudd ME 1994 Binary-encounter-dipole model for electron-impact ionization. Phys. Rev. A 50, 3954–3967. (10.1103/PhysRevA.50.3954) 9911367

[41] Gremillet L, Bonnaud G, Amiranoff F 2002 Filamented transport of laser-generated relativistic electrons penetrating a solid target. Phys. Plasmas 9, 941–948. (10.1063/1.1432994)

[42] Debayle A, Honrubia JJ, Tikhonchuk VT 2010 Divergence of laser-driven relativistic electron beams. Phys. Rev. E 82, 036405 (10.1103/PhysRevE.82.036405) 21230194

[43] Sorokovikova A, Arefiev AV, McGuffey C, Qiao B, Robinson AP, Wei MS, McLean HS, Beg FN 2016 Generation of superponderomotive electrons in multipicosecond interactions of kilojoule laser beams with solid-density plasmas. Phys. Rev. Lett. 116, 155001 (10.1103/PhysRevLett.116.155001) 27127972

[44] Iwata N, Sentoku Y, Sano T, Mima K 2019 Electron acceleration in dense plasmas heated by a picosecond relativistic laser. Nucl. Fusion 59, 086035 (10.1088/1741-4326/ab1ff9)

[45] Debayle A, Gremillet L, Honrubia JJ, D’Humières E 2013 Reduction of the fast electron angular dispersion by means of varying-resistivity structured targets. Phys. Plasmas 20, 013109 (10.1063/1.4789451)

[46] Honrubia JJ, Kaluza M, Schreiber J, Tsakiris GD, Meyer-ter-Vehn J 2005 Laser-driven fast-electron transport in preheated foil targets. Phys. Plasmas 12, 052708 (10.1063/1.1894397)

[47] Eidmann K, Meyer-ter-Vehn J, Schlegel T, Hüller S 2000 Hydrodynamic simulation of subpicosecond laser interaction with solid-density matter. Phys. Rev. E 62, 1202–1214. (10.1103/PhysRevE.62.1202) 11088579

[48] Chimier B, Tikhonchuk VT, Hallo L 2007 Heating model for metals irradiated by a subpicosecond laser pulse. Phys. Rev. B 75, 195124 (10.1103/PhysRevB.75.195124)

